# Single-cell and spatial transcriptomics integration reveals FAM49B promotes tumor-associated macrophages polarization in colorectal cancer via the MK pathway

**DOI:** 10.3389/fimmu.2025.1682637

**Published:** 2025-10-31

**Authors:** Tianyu Liu, Quchen Ding, Jin Gou, Chen Lu, Xingming Lu, Jiatong Chen, Yiming E., Lianhong Li, Chongguo Zhang, Xiaojuan Zhu, Chunzhao Yu, Xiagang Luo

**Affiliations:** ^1^ Department of General Surgery, Sir Run Run Hospital, Nanjing Medical University, Nanjing, Jiangsu, China; ^2^ Medical Centre for Digestive Diseases, The Second Affiliated Hospital of Nanjing Medical University, Nanjing, Jiangsu, China; ^3^ Department of General Surgery, The Second Affiliated Hospital of Nanjing Medical University, Nanjing, Jiangsu, China; ^4^ Department of General Surgery, The Affiliated Suzhou Hospital of Nanjing Medical University, Suzhou, Jiangsu, China; ^5^ Department of Oncology, The Second Affiliated Hospital of Nanjing Medical University, Nanjing, Jiangsu, China

**Keywords:** colorectal cancer, tumor environment (TME), FAM49B, MDK, macrophage polarization, bioinformatics

## Abstract

**Objectives:**

FAM49B has been shown to promote proliferation and metastasis of colorectal cancer (CRC) by stabilizing MYC through phosphorylation of NEK9; however, its role in shaping the immune suppressive tumor microenvironment (TME), particularly in macrophage polarization, remains unclear.

**Methods:**

We applied multi-omics approaches to study CRC by integrating 33 scRNA-seq samples from 16 CRC patients, 2 paired spatial transcriptomics (ST) samples, and bulk RNA data to characterize malignant epithelial cells (High_FAM49B_EP) and tumor-associated macrophages (TAMs). Functional validation of FAM49B was conducted via knockdown experiments and proteomics analysis.

**Results:**

A High_FAM49B_EP subpopulation was identified in primary tumors (PT) and liver metastases (LM), exhibiting elevated MYC signaling and association with poor prognosis. TAMs showed spatial heterogeneity: M1-like CXCL3^+^ TAMs predominated in PT, whereas M2-like SPP1^+^ TAMs were enriched in LM. CellChat analysis revealed that High_FAM49B_EP activated macrophage polarization through the MDK–NCL signaling axis. Pseudotime trajectory analysis confirmed differentiation from CXCL3^+^ to SPP1^+^ TAMs driven by upregulation of NCL. Spatial mapping showed co-localization of MDK^+^ epithelial cells with NCL^+^ TAMs in the immunosuppressive microenvironment. FAM49B knockdown significantly inhibited MDK expression and disrupted ECM–receptor interactions.

**Conclusions:**

FAM49B promotes immunosuppressive TME formation by mediating TAM polarization via the MDK–NCL axis, suggesting the FAM49B–MDK–NCL pathway as a potential therapeutic target for CRC metastasis.

## Introduction

Colorectal cancer (CRC) ranks as the third most commonly diagnosed malignancy worldwide and is the second leading cause of cancer-related deaths, resulting in approximately 900,000 fatalities annually (1). CRC exhibits high heterogeneity, particularly between primary tumors and liver metastases, which display marked molecular differences and divergent therapeutic responses (2, 3). Liver metastasis remains one of the principal causes of mortality in CRC patients, making a thorough understanding of the tumor immune microenvironment (TME) critical for improving treatment outcomes in patients with distant metastases (4).

In recent years, the advent of single-cell RNA sequencing (scRNA-seq) and spatial transcriptomics (ST) technologies has provided unprecedented resolution to study intratumoral heterogeneity and cellular spatial organization. While scRNA-seq dissects transcriptional profiles of diverse cell types, ST preserves the spatial context of cells within tissue architecture. These tools have been extensively utilized to uncover interactions among epithelial, immune, and stromal cells, with particular emphasis on tumor-associated macrophages (TAMs) (5–7). The polarization states of TAMs in CRC have been implicated in metastasis and immune suppression (8–12).

FAM49B is a conserved gene that has recently attracted attention in multiple cancers (13, 14). In CRC, we have demonstrated that FAM49B promotes cancer cell proliferation and migration by stabilizing c-Myc through NEK9 phosphorylation and is associated with poor patient prognosis (15). However, the heterogeneous cellular distribution of FAM49B, the existence of functional subpopulations, and its role in modulating the immune microenvironment remain unexplored systematically. Additionally, the Midkine–Nucleolin (MDK-NCL) signaling axis has been recognized as a critical mediator of communication between tumor and immune cells. MDK, a growth factor overexpressed in various solid tumors, regulates angiogenesis, cell survival, and immune suppression (16, 17). Its receptor NCL is widely expressed on macrophages and other immune cells. This signaling axis has been shown to induce regulatory T cell recruitment and M2 macrophage polarization, thereby facilitating tumor immune evasion (18).

Based on these insights, this study integrates scRNA-seq and ST analyses of 33 tumor samples—including primary tumors and liver metastases—from 16 CRC patients. We identified a malignant epithelial cell subpopulation expressing high levels of FAM49B (High_FAM49B_EP) and revealed its spatial co-localization and communication with TAMs, especially the immunosuppressive SPP1_TAMs, via the MDK-NCL pathway. Pseudotime trajectory analysis delineated the polarization progression from CXCL3_TAMs to SPP1_TAMs, and two independent spatial datasets confirmed their co-expression and functional association *in situ*. Finally, *in vitro* knockdown of FAM49B validated its role in regulating MDK expression and shaping an immunosuppressive tumor microenvironment. Collectively, this study not only maps critical communication networks within the CRC immune microenvironment but also elucidates functional coupling between specific epithelial subpopulations and immune cells, highlighting the FAM49B-MDK-NCL axis as a potential therapeutic target for metastasis inhibition and immune remodeling.

## Materials and methods

### Collection of single-cell and spatial transcriptomics data

Single-cell RNA sequencing (scRNA-seq) datasets of CRC primary tumors and liver metastases were downloaded from the Gene Expression Omnibus (GEO) database under accession number GSE245552 (19). This dataset comprises 39 CRC samples from 16 patients. For this study, 33 samples were selected for analysis, including 16 primary tumor samples and 17 liver metastasis samples. Additionally, spatial transcriptomics (ST) data of CRC were obtained from the cancerdiversity.asia database (http://www.cancerdiversity.asia/scCRLM). RNA-Seq data (in FPKM format) and corresponding clinical data for the TCGA-COAD project were retrieved from The Cancer Genome Atlas (TCGA) database. A total of 483 RNA-Seq files from CRC tissues and 41 RNA-Seq files from normal colorectal tissues were acquired.

### Quality control and annotation of single-cell data

Initially, data objects were created using the CreateSeuratObject function in Seurat (v4.4.0) with parameters min.cells = 5 and min.features = 300 (20). Doublets were filtered using DoubletFinder (v2.0.4) with a doublet rate set at 5% (21). Subsequent stringent quality control (QC) criteria were applied: (1) genes detected per cell ranged from 300 to 8000; (2) mitochondrial gene percentage did not exceed 50% of total UMIs; (3) ribosomal gene percentage did not exceed 20% of total UMIs; (4) UMI counts were greater than 1000, with the top 3% of cells by UMI count removed; (5) erythrocyte gene percentage was less than 3% of total UMIs. After these steps, 119,276 cells and 26,483 genes were retained for downstream analysis.

Batch effects across samples were corrected using Harmony (v1.2.0) (22). Clustering was performed using the FindClusters function (resolution = 0.5) based on the top 15 principal components, resulting in 19 clusters. Visualization was conducted using Uniform Manifold Approximation and Projection (UMAP). Marker genes for each cluster were identified by the FindAllMarkers function (logfc.threshold = 1). Clusters were annotated based on canonical marker genes as follows: T cells (CD3D, CD3E), myeloid cells (LYZ, CD68, CD163), epithelial cells (EPCAM, KRT20, CEACAM5), B cells (MS4A1, CD79A), fibroblasts (DCN, COL1A1, COL1A2), mast cells (TPSAB1, TPSB2, CPA3), endothelial cells (CDH5, PECAM1, CLDN5), plasma cells (JCHAIN, IGHA1, IGHA2, IGKC), and cycling cells (TOP2A, MKI67) (**Supplementary Table S1**).

### Identification of malignant epithelial cells

Copy number variation (CNV) analysis was performed on all epithelial cells using the inferCNV package (v1.3.3) (https://github.com/broadinstitute/inferCNV), with myeloid cells as the reference. Initial CNV scores per cell were calculated and visualized via heatmaps, where red and blue indicated chromosomal amplifications and deletions, respectively. The average CNV scores of myeloid and B cells were set as thresholds to exclude non-malignant epithelial cells, retaining malignant epithelial cells for further analysis.

Further clustering divided malignant cells into 14 subclusters. Cluster-specific highly expressed marker genes were identified using Seurat’s FindAllMarkers function. Biological functional features of malignant subclusters were characterized by single-sample gene set enrichment analysis (ssGSEA) using the GSVA package, based on MsigDB Hallmark gene sets. Univariate Cox regression was applied to evaluate the prognostic value of marker genes for each subcluster.

### Monocle2 pseudotime and CytoTRACE analysis

Pseudotime trajectory analysis of CRC myeloid cells was performed using Monocle (v2.30.1) (23). A single-cell dataset object was created via newCellDataSet, selecting highly variable genes based on VariableFeatures. Dimensionality reduction and trajectory inference were conducted with the DDRTree algorithm. The orderCells function assigned pseudotime values and delineated branching events to elucidate myeloid cell state transitions. Concurrently, CytoTRACE (v0.3.3) was used to compute cell stemness scores (ranging 0–1; higher scores indicate stronger stemness and lower differentiation), which were mapped onto the pseudotime trajectory to determine developmental origins (24). MK pathway activity was integrated along the timeline to visualize its dynamic changes.

### Cell–cell communication analysis

To characterize the interaction features of FAM49B-positive malignant epithelial cells, cell communication networks were inferred using the CellChat R package (v1.6.1) based on ligand-receptor interactions (25). Normalized Seurat objects were used to separately construct CellChat objects for CRC primary tumors (PT) and liver metastases (LM). Low-abundance subgroup communications were filtered using the filterCommunication function (min.cells = 10). Communication probabilities of signaling pathways were computed via computeCommunProbPathway, and aggregateNet was employed to generate aggregated communication networks, enabling comparison of network differences between PT and LM groups. Significant interactions were visualized using netVisual_circle() and netVisual_aggregate(), facilitating identification of key communication hubs. Among these, the MDK-NCL signaling pathway emerged as a core interaction axis, specifically enriched between malignant epithelial cells and myeloid cells, and was selected for subsequent spatial distribution and functional validation.

### GO and KEGG pathway enrichment analysis

Differentially expressed genes (DEGs) were identified using the FindMarkers function with parameters logfc.threshold = 1 and min.pct = 0.1. Functional annotation was performed using gseGO and gseKEGG functions in the clusterProfiler package (v4.1.0) (26). Enrichment results were visualized using the GseaVis package (v0.1.0)(https://github.com/junjunlab/GseaVis), providing an intuitive display of significantly enriched pathways and biological processes.

### Processing of CRC spatial transcriptomics data

Spatial transcriptomics data were analyzed using the Seurat package. Spots with fewer than 10 detected genes were filtered out. Variance stabilization was performed using SCTransform. Based on the top 30 principal components from RunPCA, neighborhood graphs were constructed via FindNeighbors, and spatial domains were identified using FindClusters (resolution = 0.8). Visualization was achieved by RunUMAP. To address the multicellular nature of Visium spots, spatial deconvolution was performed by integrating single-cell RNA-seq datasets with SPOTlight (v1.6.7) (27). After filtering mitochondrial and ribosomal genes, the top 3000 highly variable genes (HVGs) identified by modelGeneVar were selected. Genes with average AUC > 0.8 were used in a non-negative matrix factorization model to resolve dominant cell types per spot. Their spatial distributions were visualized using SpatialFeaturePlot.

To elucidate intercellular interaction mechanisms, spatial ligand-receptor co-localization analysis was conducted with the SpaGene package (v0.1.0) (28). This method calculates Z-scores and false discovery rates (FDR) for target gene pairs (e.g., MDK-NCL), assessing their significance relative to random spatial distribution.

### Cell lines and cell culture

Human CRC cell lines HCT116 (#FH0027), SW480 (#FH0021), and SW620 (#FH0021) were purchased from Shanghai Fuheng Biotechnology Co., Ltd. All cell lines were authenticated by short tandem repeat (STR) profiling and cultured following the supplier’s recommendations. Cells were maintained in a humidified incubator at 37°C with 5% CO_2_.

### Transient transfection and lentiviral infection

This section of methods were performed as previously described (15).

### RNA isolation and real-time quantitative polymerase chain reaction

Total RNA was isolated from each tissue and cell sample stored at -80°C using TRIzol^®^ reagent (Invitrogen, Carlsbad, CA, USA) and the SteadyPure RNA Isolation Kit (Accurate Biology, Hunan, China; Catalog Number: AG21024). The concentration and quality of the isolated RNA were evaluated using the OneDrop OD - 1000 spectrophotometer, ensuring that the A260/A280 ratio fell within the range of 1.8 - 2.0.

Subsequently, 1000 ng of total RNA was reverse - transcribed into cDNA in a 20 - μL reaction mixture using Hiscript III Reverse Transcriptase (Vazyme, Nanjing, China). The reverse - transcription reaction conditions were set as 50°C for 15 minutes followed by 85°C for 5 seconds.

In accordance with the manufacturer’s protocols, RT - qPCR was conducted using the LightCycler 480 Real - Time PCR System (Roche, Switzerland) and ChamQ Universal SYBR qPCR Master Mix (Vazyme). The sequences of the gene - specific primers are listed below:

GAPDH - Forward (F): 5′-TCAACGGATTTGGTCGTATTG-3′

GAPDH - Reverse (R): 5′-TGGGTGGAATCATATTGGAAC-3′

MDK - Forward (F): 5′-TGGAGCCGACTGCAAATACAA-3′

MDK - Reverse (R): 5′-GGCTTAGTCACGCGGATGG-3′

### DIA proteomics

HCT116 cell samples infected with either the negative control (NC) or FAM49B - KD lentivirus were separately collected. These samples were then lysed using 200 μL of an 8 M urea solution containing a protease inhibitor (Catalog Number: S8830, Sigma - Aldrich). Subsequently, the protein concentration was measured.

The extracted proteins underwent a series of sequential treatments: reduction, alkylation, and enzymatic digestion. Specifically, reduction was carried out using dithiothreitol (DTT). This was followed by alkylation modification with iodoacetamide (IAM). Finally, trypsin was added, and the mixture was incubated at 37°C overnight to complete the enzymatic digestion reaction.

The peptides obtained after digestion were desalted using a SoLAμ HRP 2 mg/mL 96 - well desalting plate (Catalog Number: 60209 - 001, Thermo Fisher Scientific). After desalting, the peptides were dried by vacuum centrifugation for subsequent analysis.

LC - MS/MS analysis was conducted on a platform that integrated the UltimateTM 3000 RSLC liquid chromatography system (Thermo Fisher Scientific) and the Q Exactive HF - X mass spectrometer (Thermo Fisher Scientific), operating in the data - independent acquisition (DIA) mode. Peptide separation was achieved through a 160 - minute gradient elution program. The mobile phase B (0.1% formic acid in 80% acetonitrile) increased from 1% to 8% within 0–4 minutes, from 8% to 30% during 4–145 minutes, from 30% to 90% in 145–150 minutes, decreased from 90% to 1% in 150–151 minutes, and was maintained at 1% for 9 minutes. The full - scan range of the mass spectrometer was set from m/z 350 to 1200, and 80 DIA windows were established for data collection.

The raw mass spectrometry data were imported into the DIA - NN software (Version 1.8.0). Targeted data extraction was performed based on a predicted human proteome database. Except for specific parameter settings, the remaining parameters were set to their default values to ensure that the false discovery rate (FDR) at both the peptide and protein levels was controlled below 1%. The protein expression intensity was finally calculated by the DIA - NN software, taking the mean of the intensities of the top three peptides.

The protein expression intensity data mentioned above were imported into the Perseus and MetaboAnalyst platforms (https://www.metaboanalyst.ca/) for statistical analysis. Initially, protein data with a missing value proportion exceeding 50% were removed. For the missing values in the remaining data, the K - nearest neighbor (KNN) algorithm was employed for imputation.

All protein expression values were log_2_ - transformed for statistical comparison. Proteins were considered differentially expressed if they met the criteria of |log_2_ fold change (FC)| > 1.5 and an adjusted p - value (FDR) < 0.05 after multiple hypothesis testing corrections. Finally, gene set enrichment analysis (GSEA) was performed on the identified differentially expressed proteins to uncover their potential biological functions and associated pathways.

### Statistical analysis

All statistical analyses and data processing in this study were performed using R (v4.3.2), Python (v3.7), and GraphPad Prism 9.0. Continuous variables with a normal distribution are presented as mean ± standard deviation (Mean ± SD), while those with non-normal (skewed) distributions are described using median and interquartile range (Median [IQR]). Statistical methods employed include independent samples t-test and Wilcoxon rank-sum test. A significance threshold of P < 0.05 was applied, with notation as follows: ns, p ≥ 0.05; *p < 0.05; **p < 0.01; ***p < 0.001.

## Result

### scRNA-seq analysis and cell type identification in CRC

**Figure 1:**
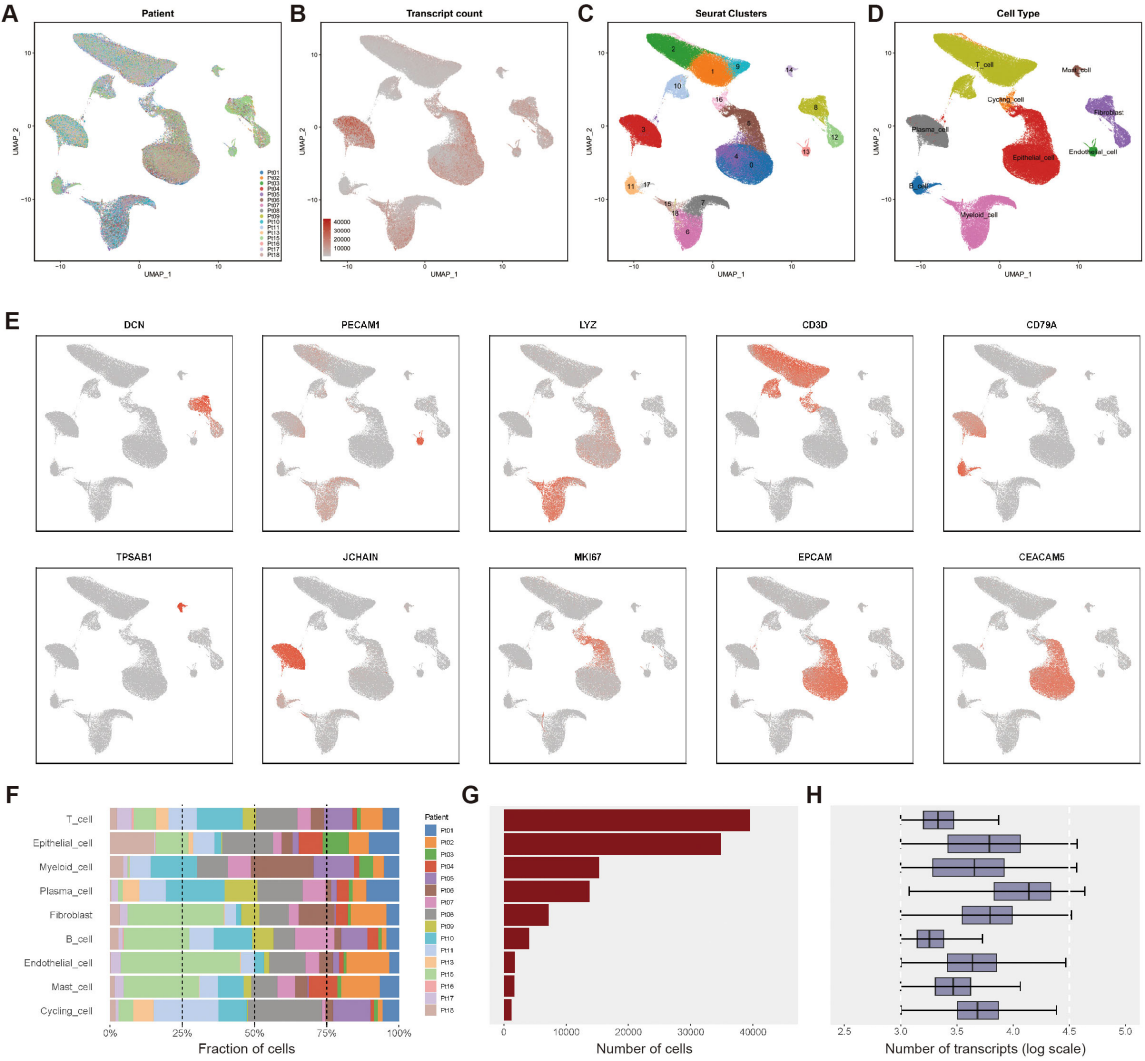
Single-cell transcriptomics atlas of CRC with PT and LM. **(A)** The UMAP plot of single - cell data colored according to the sources of 16 patients shows no significant batch effect. **(B)** The UMAP plot of transcript counts in the single - cell data set. **(C)** The Seurat clustering results for single - cell data are shown, yielding a total of 19 clusters. **(D)** Cell type annotation was performed based on the expression of marker genes, and the UMAP plots were colored according to nine major cell types. **(E)** Expression profiles of representative markers for ten distinct cell types. **(F)** The bar graph presents the distribution of the proportions of diverse cell types among different patients. **(G)** Total cell count for each identified cell type. **(H)** The log-transformed values of transcript counts for each cell type reflect the transcriptional activity at the single - cell level.

We collected single-cell RNA sequencing (scRNA-seq) data from 33 tumor samples derived from 16 CRC patients, including 16 primary tumor (PT) samples and 17 liver metastasis (LM) samples (**Figure 1A**). After stringent quality control and doublet removal, a total of 119,276 high-quality CRC cells were retained for downstream gene expression analysis. To correct for batch effects and integrate cells across patients, we applied Harmony based on patient ID, resulting in the identification of 19 distinct cell clusters (**Figure 1C**).

Based on canonical cell markers (**Figures 1B, C**), we identified nine major cell types (**Figure 1D**): epithelial cells (34,876 cells), B cells (4,040 cells), proliferating cells (1,183 cells), endothelial cells (1,747 cells), fibroblasts (7,208 cells), mast cells (1,644 cells), plasma cells (13,745 cells), myeloid cells (15,309 cells), and T cells (39,524 cells). The expression levels of representative marker genes for each cell type are shown in **Figure 1E**. The proportions of these cell types across samples are displayed in **Figure 1F**, the absolute cell counts in **Figure 1G**, and detailed transcript counts for each cell type in **Figure 1H**.

### Identification of FAM49B-associated malignant epithelial cells

**Figure 2:**
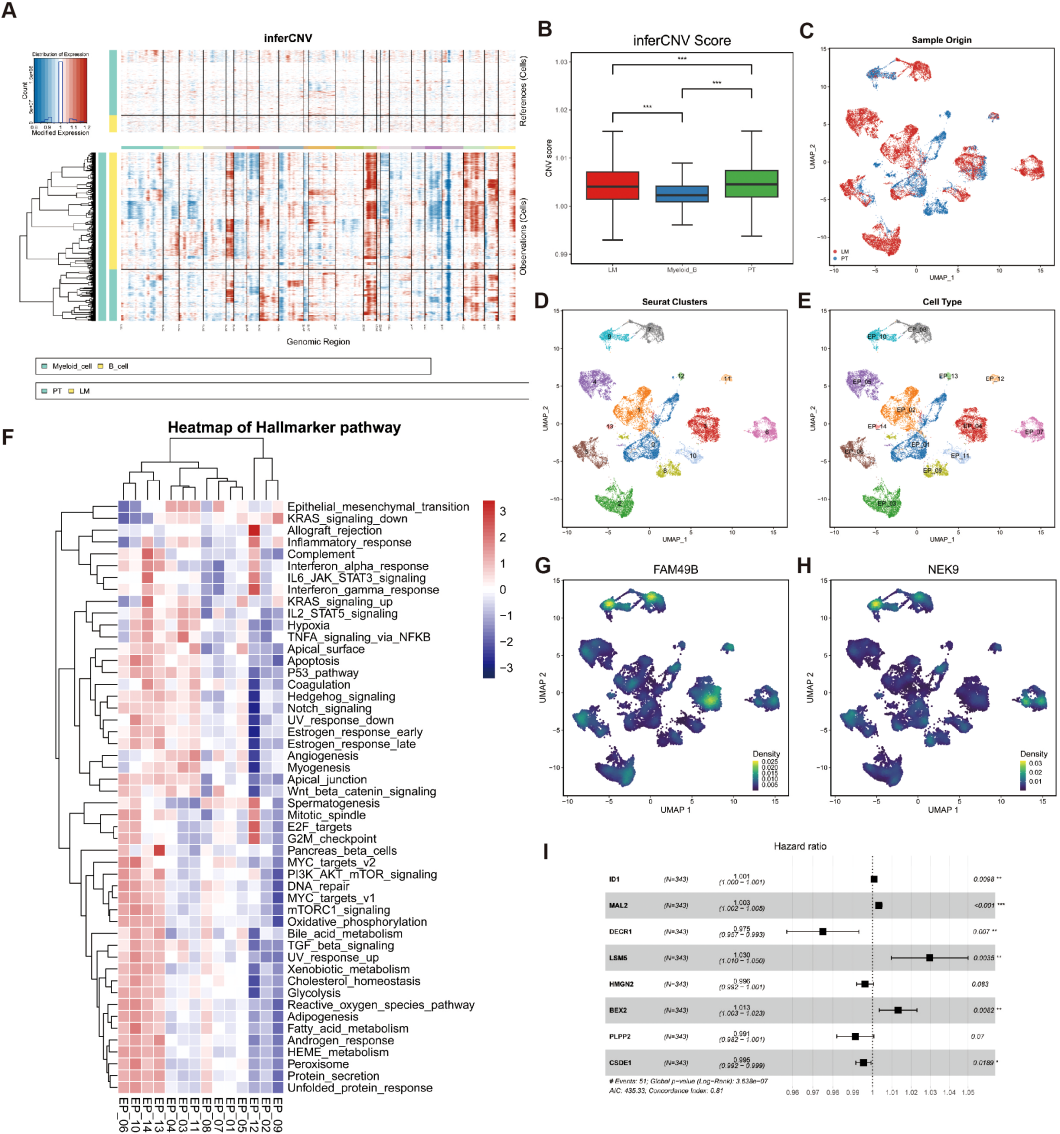
Identification of malignant epithelial cells. **(A)** Chromosomal heatmaps of CNVs in epithelial cells were inferred with reference to myeloid cells and B cells, where red indicates amplification and blue indicates deletion. **(B)** Boxplot for CNV Scores (PT, LM myeloid cells and B cells). *p < 0.05, **p < 0.01, and ***p < 0.001, Student’s t-test. **(C)** UMAP plots of malignant epithelial cells colored by sample origin (PT and LM). **(D)** UMAP plots of malignant epithelial cells, with 14 clusters. **(E)** The UMAP plot shows that malignant epithelial cells are divided into 14 subgroups. **(F)** Heatmap showing GSVA scores of hallmarker gene sets for 14 malignant epithelial cell subtypes. **(G)** The UMAP plot shows the main distribution areas of the FAM49B gene. **(H)** The UMAP plot shows the main distribution areas of the NEK9 gene. **(I)** Univariate Cox analysis of key marker genes in the High_FAM49B_EP group showed their prognostic significance through hazard ratios, confidence intervals, and P values.

To identify malignant epithelial cells characterized by FAM49B expression, we applied InferCNV to calculate copy number variation (CNV) scores for each epithelial cell, using myeloid and B cells as reference populations (**Figure 2A**). The resulting chromosomal alteration heatmap, annotated by tissue origin, revealed distinct CNV patterns between primary tumors (PT) and liver metastases (LM). Notably, CNV scores were higher in PT-derived epithelial cells than in those from LM, indicating substantial epithelial heterogeneity between the two sites (**Figure 2B**).

We used the mean CNV score of the myeloid and B cell populations as a threshold to classify epithelial cells into malignant (22,780 cells) and non-malignant (12,096 cells) categories. Among these, 7,293 malignant epithelial cells originated from PT samples, while 15,487 were from LM samples (**Figure 2C**). Further clustering analysis identified 11 malignant epithelial subpopulations (**Figures 2D, E**).

To explore the heterogeneity among these malignant epithelial subgroups, we performed Gene Set Variation Analysis (GSVA) using the Hallmark gene sets (**Figure 2F**). Among all clusters, EP_10 exhibited the highest enrichment score for the MYC_targets_v2 pathway, consistent with our previous findings. We also examined the expression patterns of FAM49B and NEK9 across malignant epithelial subtypes and found that both genes were significantly co-localized in EP_08 and EP_10 clusters (**Figures 2G, H**), further supporting our earlier conclusions. These two clusters were defined as the High_FAM49B_EP subpopulation. Finally, univariate Cox regression analysis identified prognostically unfavorable genes within the High_FAM49B_EP group (**Figure 2I**).

### Identification of macrophage subpopulations

**Figure 3:**
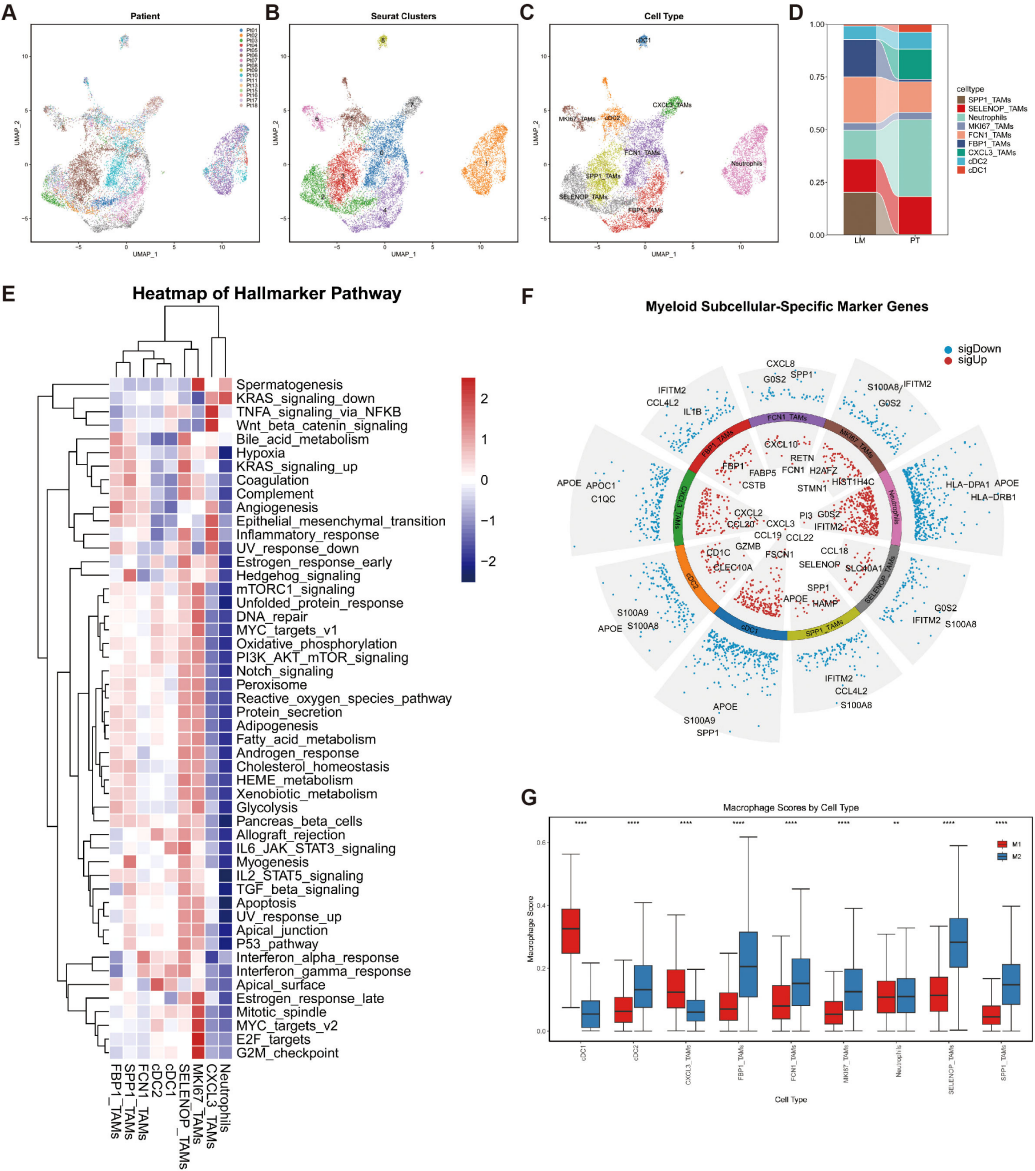
Phenotypic identification of myeloid cells. **(A)** UMAP plots of myeloid cells colored by patient ID. **(B)** UMAP plots of myeloid cells colored according to Seurat clusters. **(C)** UMAP plots of myeloid cells colored by cell type. **(D)** Bar chart depicting the differences in myeloid cell proportions between the PT and LM groups. **(E)** Heatmap displaying GSVA scores of hallmark gene sets across nine myeloid cell subtypes. **(F)** Bar plots presenting the top three upregulated and downregulated genes in nine myeloid cell subsets. **(G)** Box plots illustrating gene set scores for M1 and M2 polarization in nine myeloid cell subtypes.

We performed subclustering analysis on myeloid cells (**Figure 3A**), resulting in the identification of nine distinct subpopulations (**Figure 3B**). Among these, macrophage clusters were annotated based on their predominant marker gene expression, including SPP1_TAMs, SELENOP_TAMs, MKI67_TAMs, FCN1_TAMs, FBP1_TAMs, and CXCL3_TAMs (**Figures 3C, F**).

Comparative analysis revealed compositional differences in macrophage subtypes between primary tumors (PT) and liver metastases (LM). CXCL3_TAMs were predominant in PT samples but markedly reduced in LM, whereas SPP1_TAMs and FBP1_TAMs were more abundant in LM (**Figure 3D**).

To further explore pathway-level characteristics of each myeloid subpopulation, we conducted Gene Set Variation Analysis (GSVA) using the Hallmark gene sets (**Figure 3E**). FBP1_TAMs exhibited enrichment in Hypoxia, Bile Acid Metabolism, and Angiogenesis pathways, which are closely associated with tumor immune suppression. SPP1_TAMs were significantly enriched in pathways such as KRAS signaling up, Coagulation, and Hedgehog signaling, suggesting a potential role in promoting tumor vascularization. In contrast, CXCL3_TAMs showed elevated scores in the Inflammatory Response pathway.

These enrichment profiles suggest that SPP1_TAMs and FBP1_TAMs may correspond to M2-like macrophages, whereas CXCL3_TAMs are more aligned with M1-like phenotypes. To further quantify the polarization states of these macrophage subtypes, we curated gene sets associated with M1 and M2 macrophage phenotypes and calculated signature scores for each subpopulation (**Supplementary Table S2**). The results showed that CXCL3_TAMs had significantly higher scores for the M1-associated gene set, while SPP1_TAMs and FBP1_TAMs were more enriched in the M2-associated gene set, further validating the GSVA-based findings.

### The MK signaling pathway could drive macrophage polarization

**Figure 4:**
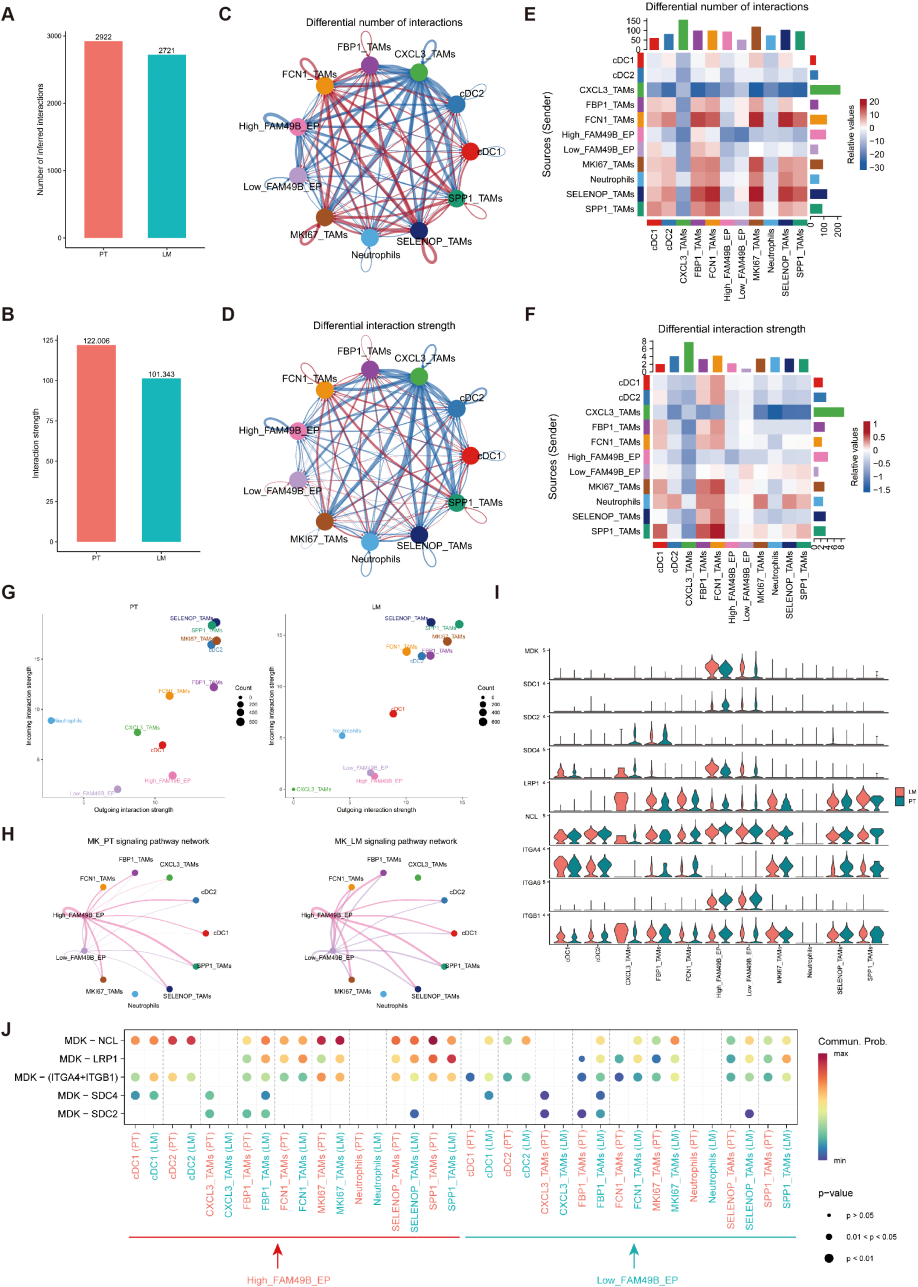
Intercellular communication of myeloid cells and malignant EPs. **(A, B)** Bar graphs depict the quantity and intensity of intercellular interactions within PT and LM. **(C, D)** Illustrations of the quantity **(C)** and intensity **(D)** of intercellular interactions are presented. Herein, the size of each dot is proportional to the cell number, and the thickness of each line corresponds proportionally to the quantity or intensity of the interactions. **(E, F)** Heatmaps unveil the alterations in the quantity **(E)** and intensity **(F)** of intercellular interactions between PT and LM. **(G)** The communication signal strength between myeloid cells and malignant EPs in the PT and LM groups was analyzed via CellChat. **(H)** A circular plot depicts the inferred MK signaling network in PT and LM. **(I)** A violin plot presents the expression levels of nine genes associated with the MK signaling network. **(J)** A bubble plot illustrates the communication status of MK pathway-specific ligand–receptor pairs between malignant epithelial cells and myeloid cells at different FAM49B expression levels in PT and LM. The size of each dot indicates the P - value, while the color represents the communication probability.

To further investigate how High_FAM49B_EP cells regulate macrophages within the tumor microenvironment (TME), we employed the CellChat tool to analyze intercellular communication. The results revealed that both the number and strength of interactions among major cell types were generally higher in primary tumors (PT) than in liver metastases (LM), although the differences were relatively modest (**Figures 4A–D**).

Differential interaction heatmaps between PT and LM indicated that CXCL3_TAMs exhibited the most pronounced changes in both the number and strength of interactions. In comparison with other epithelial subpopulations, High_FAM49B_EP cells also showed more prominent changes in interaction patterns (**Figures 4E, F**).

We next evaluated the outgoing and incoming signaling intensities for each myeloid subcluster. CXCL3_TAMs showed stronger outgoing and incoming signaling activity in PT. High_FAM49B_EP cells exhibited increased outgoing signals specifically in PT, while FBP1_TAMs received more signals in LM. SPP1_TAMs displayed active signaling behavior in both PT and LM (**Figure 4G**).

Notably, High_FAM49B_EP cells primarily communicated with macrophages through the MK (midkine) signaling pathway, with this interaction being more pronounced in PT samples. As shown in **Figure 4H**, the MK pathway signaling network revealed High_FAM49B_EP as the main sender population and myeloid cells as the predominant receivers. Within this pathway, MDK was highly expressed in malignant epithelial cells, whereas NCL was broadly expressed in macrophages (**Figure 4I**).

We further analyzed the MDK–NCL interaction between malignant epithelial cells and macrophages, which revealed a significantly strong interaction (**Figure 4J**). These findings highlight the critical role of the MDK–NCL axis in shaping the tumor microenvironment and suggest a potential mechanism by which High_FAM49B_EP cells could drive macrophage polarization.

### Polarization trajectory from CXCL3_TAMs to SPP1_TAMs

**Figure 5:**
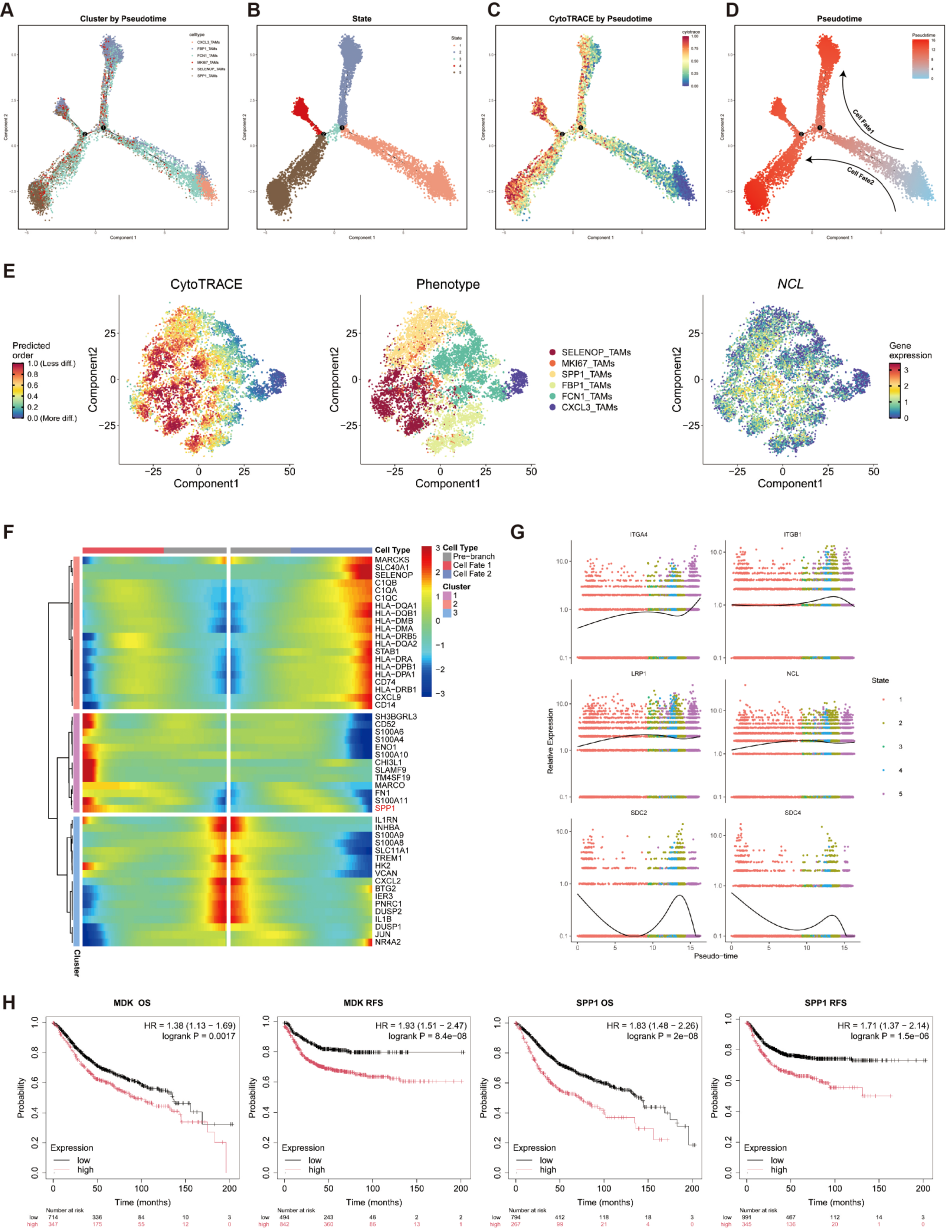
Pseudotime analysis was employed to delineate the differentiation trajectory from CXCL3_TAMs to SPP1_TAMs. **(A)** Pseudotime trajectory analysis reveals the differentiation trajectories of six cell types. **(B)** Pseudotime trajectory analysis reveals five distinct cellular differentiation states. **(C)** Projection of CytoTRACE scores onto the Pseudotime trajectory. **(D)** Pseudotime scores are mapped along the cellular differentiation trajectory. **(E)** CytoTRACE-reconstructed differentiation trajectories of TAMs (left to right): CytoTRACE scores, distribution of six TAM subsets, and NCL expression. **(F)** The heatmap shows the relative expression changes of differentially expressed genes driving differentiation toward cell fates 1 and 2 in TAMs clusters classified into three groups based on pseudo-temporal clustering. **(G)** Expression dynamics of MK pathway genes along the pseudotime trajectory. **(H)** Kaplan-Meier curves illustrate the impact of MDK and SPP1 expression on CRC patient OS and RFS.

To delineate the evolutionary dynamics of macrophages in CRC, we constructed a pseudotime developmental trajectory based on single-cell RNA-seq data (**Figure 5A**). This trajectory classified macrophages into five developmental states (**Figure 5B**), thereby outlining their differentiation path within the CRC tumor microenvironment.

By integrating CytoTRACE scores to assess differentiation potential, we observed that the CXCL3_TAMs cluster—positioned at the bottom right of the trajectory—exhibited the lowest differentiation potential, identifying it as the likely origin of the macrophage developmental path (**Figures 5C, D**). Furthermore, two distinct differentiation trajectories (designated as cellfate1 and cellfate2) were identified. The CXCL3_TAMs cluster, characterized as M1-like macrophages, served as the starting point for differentiation towards M2-like macrophages. This shift towards a more immunosuppressive and tumor-promoting macrophage phenotype significantly contributes to the establishment of an immunosuppressive tumor microenvironment.

Further analysis of CytoTRACE scores revealed that NCL, the receptor of the MK signaling pathway, was predominantly enriched in M2-like macrophages (**Figure 5E**). Differential gene expression analysis at Branch Point 1 indicated significantly higher expression of SPP1 in CellFate1, suggesting a transition trajectory from CXCL3_TAMs toward SPP1_TAMs (**Figure 5F**).

Lastly, we examined the expression dynamics of MK pathway-related genes along the pseudotime trajectory. The results showed a progressive increase in NCL expression as pseudotime advanced (**Figure 5G**), further supporting its role in driving the polarization of CXCL3_TAMs toward the M2-like SPP1_TAM phenotype.

Consistently, survival analysis also demonstrated that high expression levels of both MDK and SPP1 were significantly associated with poorer overall survival (OS) and relapse-free survival (RFS) (**Figure 5H**), indicating that MDK-driven macrophage polarization promotes an immunosuppressive tumor microenvironment and is closely linked to adverse clinical outcomes.

### Spatial distribution characteristics of CXCL3_TAMs and SPP1_TAMs

**Figure 6:**
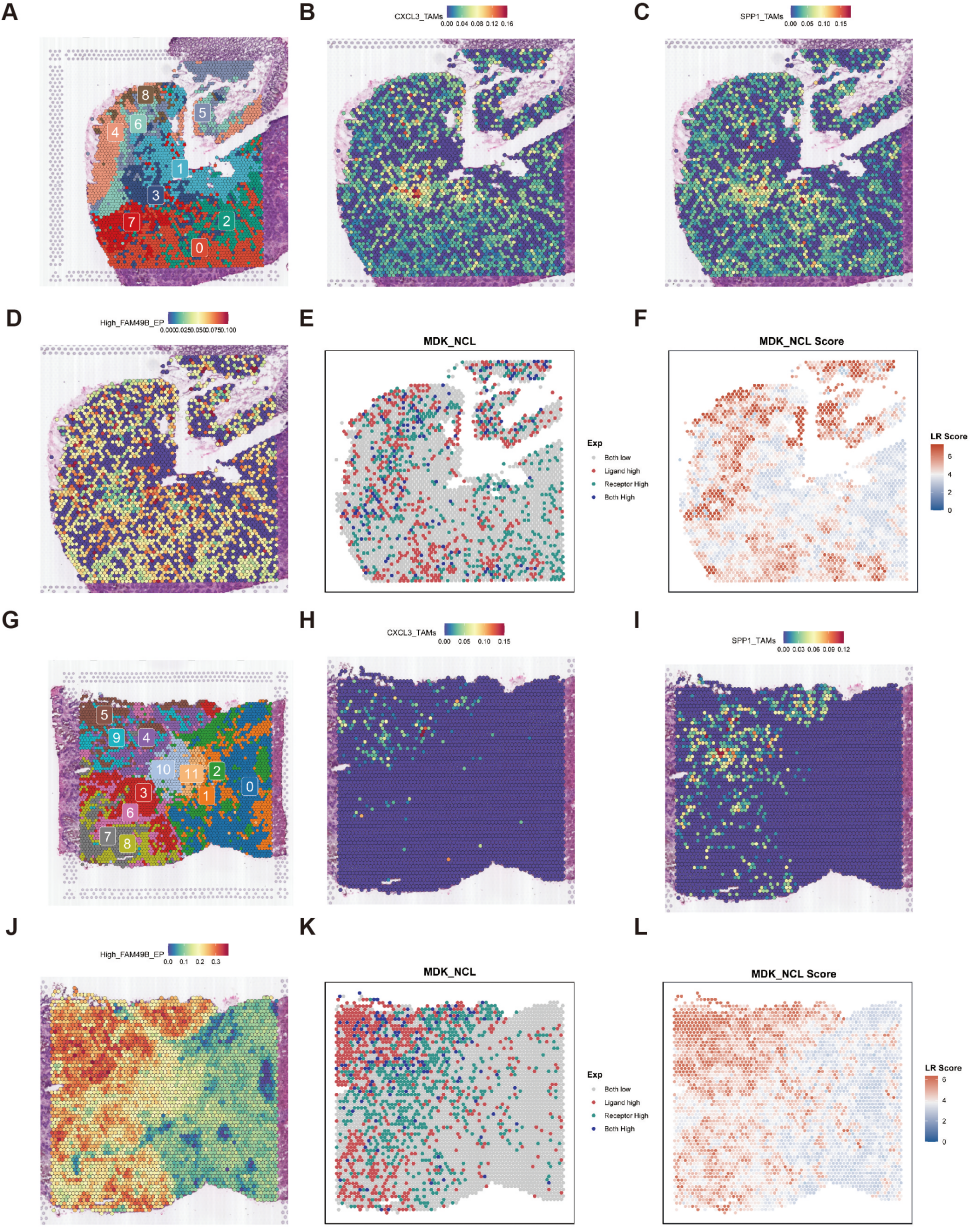
Spatial transcriptomic slices of primary colorectal cancer lesions. **(A)** Cluster plot of 0–8 subgroups clustered by Seurat. **(B–D)** Spatial plot showing the expression of CXCL3_TAMs, SPP1_TAMs, and High_FAM49B_EP in PT predicted by SPOTlight. **(E, F)** Spatial mapping of the MDK ligand, NCL receptor, and their binding score in the MDK-NCL ligand-receptor interaction analysis in PT. **(G)** Cluster plot of 0–11 subgroups clustered by Seurat. **(H–J)** Spatial plot showing the expression of CXCL3_TAMs, SPP1_TAMs, and High_FAM49B_EP in LM predicted by SPOTlight. **(K, L)** Spatial mapping of the MDK ligand, NCL receptor, and their binding score in the MDK-NCL ligand-receptor interaction analysis in LM.

To elucidate the spatial organization of High_FAM49B_EP, CXCL3_TAMs, and SPP1_TAMs, we conducted a study based on spatial transcriptomics (ST) data from two CRC liver metastasis patients. In the first sample, tissue spots were clustered into 0–8 spatial clusters using Louvain clustering (**Figure 6A**), and the cell-type signatures defined by scRNA-seq were projected onto the ST spots using the SPOTlight tool (**Figures 6B–D**). The results revealed that CXCL3_TAMs and SPP1_TAMs co-localized within cluster 3. Concurrently, SpaGene detected high expression of the ligand MDK and receptor NCL in this region, confirming that these two cell types form a spatial interaction network via the MDK-NCL signaling axis (**Figure 6E**). In the paired liver metastasis samples, tissue spots were clustered into 0–11 spatial clusters(**Figure 6G**). The results of SPOTlight deconvolution analysis indicated that CXCL3_TAMs and SPP1_TAMs were significantly enriched in clusters 4, 5, and 9, showing a high degree of consistency with the spatial localization of High_FAM49B_EP (**Figures 6H–J**). Additionally, SpaGene confirmed the co - localization of MDK and NCL within these enriched regions, further corroborating the role of the MDK - NCL signaling axis in spatial interactions (**Figures 6K, L**).

We performed a similar analysis on a second sample to validate our findings. Following Louvain clustering of tissue spots into 0–12 clusters, SPOTlight deconvolution revealed significant enrichment of CXCL3_TAMs and SPP1_TAMs in clusters 4, 5, 8, 10, and 11(**Supplementary Figures S1A–D**). SpaGene further confirmed the co-localization of MDK and NCL within these regions (**Supplementary Figures S1E, F**). Comparable patterns were detected in the liver metastatic foci of this sample (**Supplementary Figures S1G–L**). The cross-sample consistency supports the conclusion that High_FAM49B_EP drives the differentiation of CXCL3_TAMs into SPP1_TAMs via the MDK-NCL signaling pathway, thereby contributing to the remodeling of the immunosuppressive tumor microenvironment. These findings were independently validated in two paired samples.

### Validation of MDK expression after FAM49B knockdown

**Figure 7:**
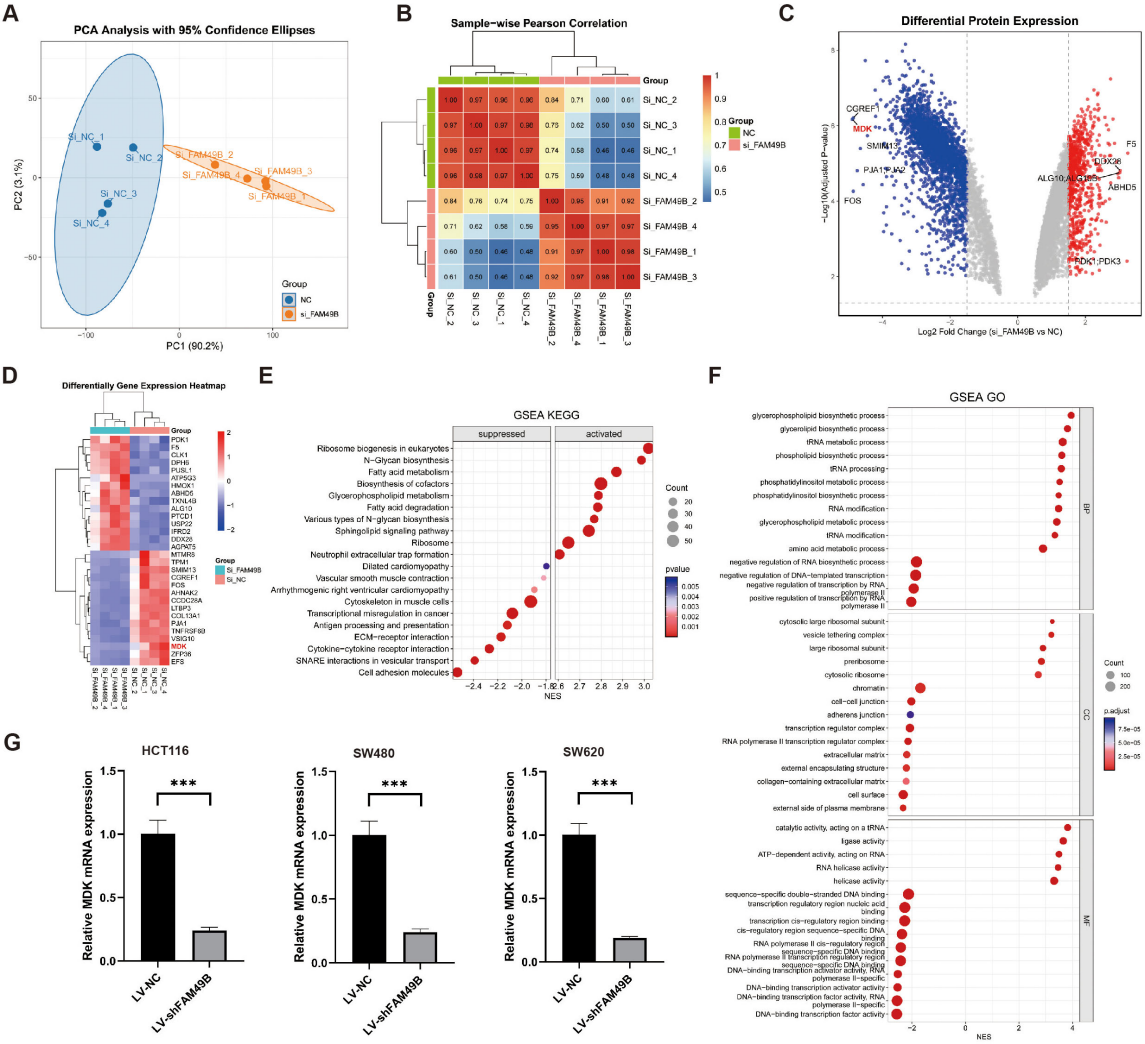
FAM49B knockdown suppresses MDK expression. **(A)** PCA was performed on si_FAM49B and si_NC in the HCT116 cell line. **(B)** Heatmap of correlations between si_FAM49B and si_NC samples. **(C)** Volcano plot of differentially expressed genes between si_FAM49B and si_NC. **(D)** Heatmap of the top 15 differentially expressed genes between si_FAM49B and si_NC. **(E)** GSEA KEGG enrichment analysis of differentially expressed genes between si_FAM49B and si_NC. **(F)** GSEA GO enrichment analysis of differentially expressed genes between si_FAM49B and si_NC. **(G)** The relative expression of MDK mRNA was analyzed via RT-qPCR in CRC cells transfected with LV_FAM49B and LV_NC lentiviruses.

To investigate the function of FAM49B, we knocked down its expression in the human CRC cell line HCT116 using specific siRNA. After establishing a stable knockdown model (si_FAM49B), proteomic analysis was performed on both si_FAM49B and control si_NC cells (**Supplementary Table S3**). PCA analysis and sample correlation heatmaps confirmed significant differences between the si_FAM49B and si_NC groups(**Figures 7A, B**). Differential protein screening (|Log_2_FC| > 1.5 and p < 0.05) revealed that MDK expression was significantly downregulated following FAM49B knockdown, which was corroborated by both the volcano plot and the top 15 differential protein heatmap (**Figures 7C, D**).

GSEA analysis of differential proteins indicated that, in KEGG enrichment, ribosome biogenesis, N-glycan biosynthesis, and fatty acid metabolism pathways were significantly upregulated, while ECM-receptor interaction, cytokine-cytokine receptor interaction, SNARE interactions, and pathways involving cell adhesion molecules were markedly downregulated (**Figure 7E**). In GO enrichment, phospholipid-related biosynthetic and metabolic pathways, including glycerophospholipid and phosphatidylinositol biosynthesis, were significantly upregulated, whereas pathways associated with cell adhesion, such as intercellular and homophilic adhesion mediated by plasma membrane adhesion molecules, were significantly downregulated (**Figure 7F**).

These proteomic results indicate that knockdown of FAM49B significantly suppresses MDK expression and affects multiple lipid metabolism and biosynthesis pathways. Moreover, the critical downregulation of ECM-receptor interaction and cell adhesion molecule pathways suggests a potential weakening of tumor cell interactions with the extracellular matrix (ECM) and intercellular communication. Furthermore, in three CRC-derived cell models—HCT116, SW480, and SW620—FAM49B gene knockdown significantly inhibited MDK mRNA expression (p < 0.001), indicating that this regulatory relationship is consistent across different cell lines (**Figure 7G**).

## Discussion

In this study, we systematically investigated the role of High_FAM49B_EP in promoting macrophage polarization through the MDK-NCL signaling axis in CRC by integrating single-cell RNA sequencing (scRNA-seq) and spatial transcriptomics. Our findings reveal that the MDK-NCL pathway contributes to immunosuppression and tumor immune evasion mechanisms, providing novel insights into the potential of targeting this axis as a therapeutic strategy. This work deepens our understanding of tumor microenvironment (TME) remodeling in CRC.

In our previous research, FAM49B was shown to activate NEK9 phosphorylation, stabilize and activate c-Myc, and its expression correlated closely with patient prognosis (15). However, the mechanisms by which FAM49B shapes the immune microenvironment in CRC remained unclear. Our current study identifies the MDK-NCL signaling axis as a central mediator in the interaction between High_FAM49B_EP and tumor-associated macrophages (TAMs). MDK, a multifunctional growth factor, is highly expressed in High_FAM49B_EP cells, while its receptor NCL is broadly distributed on macrophage surfaces (29–31). This ligand-receptor specificity forms the molecular basis for intercellular communication. Notably, NCL expression is significantly higher in SPP1_TAMs compared to CXCL3_TAMs, which may be a key driver of macrophage phenotypic transition. Upon MDK binding to NCL, macrophages are induced to polarize from an M1 toward an immunosuppressive M2 phenotype (32). This finding echoes the work of Yu Fu et al., whose single-cell and spatial transcriptomics analyses also revealed that MDK-NCL promotes the formation of an immunosuppressive microenvironment in lung adenocarcinoma (LUAD), with high MDK-NCL expression associated with increased infiltration of myeloid-derived suppressor cells (MDSCs) and M2-like macrophages (18, 33, 34).

Accumulating studies have demonstrated that MDK signaling promotes the polarization of tumor-associated macrophages (TAMs) toward an immunosuppressive M2 phenotype. In glioma, MDK secreted by GBM cells drives macrophage polarization toward the M2 phenotype by activating receptors on macrophages, leading to the secretion of cytokines such as CXCL1 and thereby fostering an immunosuppressive environment (35). Similarly, in gallbladder cancer, upregulation of MDK enhances its interaction with LRP1—expressed by tumor-infiltrating macrophages—promoting the differentiation of immunosuppressive macrophages (31). The MDK-NCL axis has been recognized as a promising therapeutic target, as it can be targeted by monoclonal antibodies or small molecule inhibitors to reverse immunosuppression (36). Strategies aimed at reprogramming immunosuppressive myeloid cells, including macrophages influenced by MDK, can shift the TME from an immunosuppressive to an immunostimulatory state (37). Studies in melanoma models have demonstrated that genetically targeting MDK can overcome resistance to PD-1/PD-L1 inhibitors and enhance therapeutic efficacy (38). Targeting this axis is thus identified as a potential strategy to reprogram the TME, suppress macrophage-mediated immunosuppression, and synergize with immune checkpoint inhibitors.

A major innovation of this study is the linkage of FAM49B expression to MDK secretion. Proteomic data demonstrate that FAM49B knockdown significantly inhibits MDK expression, suggesting that FAM49B may regulate MDK synthesis at the transcriptional or post-transcriptional level. We propose the existence of a FAM49B-MDK-NCL regulatory cascade. This pathway potentially explains why High_FAM49B_EP exhibits stronger immunomodulatory capacity. In liver metastatic lesions, although the proportion of High_FAM49B_EP cells is low, SPP1_TAMs are significantly increased, likely due to the specific selective pressures of the metastatic microenvironment—characterized by hypoxia, elevated lactate, and bile acid—which favor M2 macrophage polarization, consistent with the enrichment of FBP1_TAMs in bile acid metabolic pathways (39–41).

The critical role of SPP1+ macrophages in CRC progression has been highlighted in multiple studies (42–46). Here, we observed significant enrichment of SPP1_TAMs in liver metastases and the highest scores within M2 polarization gene sets. These macrophages directly promote tumor metastasis by secreting immunosuppressive factors (e.g., IL-10, TGF-β) and pro-angiogenic factors (e.g., VEGF) (47, 48). Importantly, spatial analysis revealed that SPP1_TAMs are spatially adjacent to malignant cells, forming a microenvironment conducive to tumor invasion and survival. This spatial distribution correlates with poor prognosis in patients with liver metastases and aligns with previous findings that SPP1+ macrophage enrichment significantly associates with reduced survival in CRC patients (49–51).

Despite comprehensively delineating the role of the FAM49B-MDK-NCL axis in the CRC immune microenvironment, several limitations and future directions remain. First, sample heterogeneity is a constraint: although we integrated 33 samples from 16 patients, spatial transcriptomics was performed only on two liver metastasis cases. Expanding sample size is needed to validate the generalizability of this signaling pathway. Moreover, incorporating a broader range of clinical stages and molecular subtypes will allow exploration of axis activity variations across subtypes (52).

In summary, through integrated multi-omics analysis, this study is the first to elucidate the complete mechanism by which FAM49B-positive epithelial cells promote macrophage M2 polarization via the MDK-NCL signaling axis. This discovery not only advances understanding of the heterogeneity formation mechanisms within the CRC immune microenvironment but also offers a novel therapeutic target to overcome immune therapy resistance. Future studies should validate the universality of this axis in larger clinical cohorts and further investigate its interplay with tumor metabolic microenvironment. Targeting the FAM49B-MDK-NCL pathway, particularly in combination with existing immune checkpoint inhibitors, holds promise as a new avenue for precision immunotherapy in CRC, ultimately improving survival outcomes for patients with metastatic disease.

## Data Availability

The datasets presented in this study can be found in online repositories. The names of the repository/repositories and accession number(s) can be found in the article/**Supplementary Material**.

## References

[1] Siegel RL Miller KD Fuchs HE Jemal A. Cancer statistics, 2022. CA Cancer J Clin. (2022) 72:7–33. doi: 10.3322/caac.21708, PMID: 35020204

[2] Wang F Long J Li L Wu ZX Da TT Wang XQ. Single-cell and spatial transcriptome analysis reveals the cellular heterogeneity of liver metastatic colorectal cancer. Sci Adv. (2023) 9:eadf5464. doi: 10.1126/sciadv.adf5464, PMID: 37327339 PMC10275599

[3] Li R Liu X Huang X Zhang D Chen Z Zhang J. Single-cell transcriptomic analysis deciphers heterogenous cancer stem-like cells in colorectal cancer and their organ-specific metastasis. Gut. (2024) 73:470–84. doi: 10.1136/gutjnl-2023-330243, PMID: 38050068 PMC10894846

[4] Wang Y Zhong X He X Hu Z Huang H Chen J. Liver metastasis from colorectal cancer: pathogenetic development, immune landscape of the tumour microenvironment and therapeutic approaches. J Exp Clin Cancer Res. (2023) 42:177. doi: 10.1186/s13046-023-02729-7, PMID: 37480104 PMC10362774

[5] Schaer DJ Schulthess-Lutz N Baselgia L Hansen K Buzzi RM Humar R. Hemorrhage-activated NRF2 in tumor-associated macrophages drives cancer growth, invasion, and immunotherapy resistance. J Clin Invest. (2023) 134(3). doi: 10.1172/JCI174528, PMID: 38060331 PMC10849758

[6] Veschi V Verona F Di Bella S Turdo A Gaggianesi M Di Franco S. C1Q(+) TPP1(+) macrophages promote colon cancer progression through SETD8-driven p53 methylation. Mol Cancer. (2025) 24:102. doi: 10.1186/s12943-025-02293-y, PMID: 40165182 PMC11956498

[7] Wu Y Yang S Ma J Chen Z Song G Rao D. Spatiotemporal immune landscape of colorectal cancer liver metastasis at single-cell level. Cancer Discov. (2022) 12:134–53. doi: 10.1158/2159-8290.CD-21-0316, PMID: 34417225

[8] Stadler M Pudelko K Biermeier A Walterskirchen N Gaigneaux A Weindorfer C. Stromal fibroblasts shape the myeloid phenotype in normal colon and colorectal cancer and induce CD163 and CCL2 expression in macrophages. Cancer Lett. (2021) 520:184–200. doi: 10.1016/j.canlet.2021.07.006, PMID: 34256095

[9] Xu H Li S Liu Y Sung YY Zhou Y Wu H. A novel pH-sensitive nanoparticles encapsulating anti-PD-1 antibody and MDK-siRNA overcome immune checkpoint blockade resistance in HCC via reshaping immunosuppressive TME. J Exp Clin Cancer Res. (2025) 44:148. doi: 10.1186/s13046-025-03396-6, PMID: 40380202 PMC12082952

[10] Huang C Ou R Chen X Zhang Y Li J Liang Y. Tumor cell-derived SPON2 promotes M2-polarized tumor-associated macrophage infiltration and cancer progression by activating PYK2 in CRC. J Exp Clin Cancer Res. (2021) 40:304. doi: 10.1186/s13046-021-02108-0, PMID: 34583750 PMC8477524

[11] Zhao S Mi Y Guan B Zheng B Wei P Gu Y. Tumor-derived exosomal miR-934 induces macrophage M2 polarization to promote liver metastasis of colorectal cancer. J Hematol Oncol. (2020) 13:156. doi: 10.1186/s13045-020-00991-2, PMID: 33213490 PMC7678301

[12] He X Chen H Zhong X Wang Y Hu Z Huang H. BST2 induced macrophage M2 polarization to promote the progression of colorectal cancer. Int J Biol Sci. (2023) 19:331–45. doi: 10.7150/ijbs.72538, PMID: 36594082 PMC9760448

[13] Li Y Xiong Y Wang Z Han J Shi S He J. FAM49B promotes breast cancer proliferation, metastasis, and chemoresistance by stabilizing ELAVL1 protein and regulating downstream Rab10/TLR4 pathway. Cancer Cell Int. (2021) 21:534. doi: 10.1186/s12935-021-02244-9, PMID: 34645466 PMC8513284

[14] Huang H Tsui YM Ho DW Chung CY Sze KM Lee E. LANCL1, a cell surface protein, promotes liver tumor initiation through FAM49B-Rac1 axis to suppress oxidative stress. Hepatology. (2024) 79:323–40. doi: 10.1097/HEP.0000000000000523, PMID: 37540188 PMC10789379

[15] Lu C Liu T Yimin E Miao L Yu C Zhang J. FAM49B drives colorectal cancer progression by stabilizing c-Myc through NEK9 phosphorylation. Biofactors. (2025) 51:e2158. doi: 10.1002/biof.2158, PMID: 39780509

[16] Carvalho RF do Canto LM Abildgaard C Aagaard MM Tronhjem MS Waldstrøm M. Single-cell and bulk RNA sequencing reveal ligands and receptors associated with worse overall survival in serous ovarian cancer. Cell Commun Signal. (2022) 20:176. doi: 10.1186/s12964-022-00991-4, PMID: 36352420 PMC9648056

[17] Yu X Xie L Ge J Li H Zhong S Liu X. Integrating single-cell RNA-seq and spatial transcriptomics reveals MDK-NCL dependent immunosuppressive environment in endometrial carcinoma. Front Immunol. (2023) 14:1145300. doi: 10.3389/fimmu.2023.1145300, PMID: 37081869 PMC10110842

[18] Fu Y Li S Zhao Y Zhang X Mao X Xu R. Integrative single-cell and spatial transcriptomics analysis reveals MDK-NCL pathway’s role in shaping the immunosuppressive environment of lung adenocarcinoma. Front Immunol. (2025) 16:1546382. doi: 10.3389/fimmu.2025.1546382, PMID: 40396179 PMC12089103

[19] Liu X Wang X Yang Q Luo L Liu Z Ren X. Th17 cells secrete TWEAK to trigger epithelial-mesenchymal transition and promote colorectal cancer liver metastasis. Cancer Res. (2024) 84:1352–71. doi: 10.1158/0008-5472.CAN-23-2123, PMID: 38335276

[20] Hao Y Hao S Andersen-Nissen E Mauck WM Zheng S Butler A. Integrated analysis of multimodal single-cell data. Cell. (2021) 184:3573–87.e29. doi: 10.1016/j.cell.2021.04.048, PMID: 34062119 PMC8238499

[21] McGinnis CS Murrow LM Gartner ZJ. DoubletFinder: doublet detection in single-cell RNA sequencing data using artificial nearest neighbors. Cell Syst. (2019) 8:329–37.e4. doi: 10.1016/j.cels.2019.03.003, PMID: 30954475 PMC6853612

[22] Korsunsky I Millard N Fan J Slowikowski K Zhang F Wei K. Fast, sensitive and accurate integration of single-cell data with Harmony. Nat Methods. (2019) 16:1289–96. doi: 10.1038/s41592-019-0619-0, PMID: 31740819 PMC6884693

[23] Qiu X Mao Q Tang Y Wang L Chawla R Pliner HA. Reversed graph embedding resolves complex single-cell trajectories. Nat Methods. (2017) 14:979–82. doi: 10.1038/nmeth.4402, PMID: 28825705 PMC5764547

[24] Gulati GS Sikandar SS Wesche DJ Manjunath A Bharadwaj A Berger MJ. Single-cell transcriptional diversity is a hallmark of developmental potential. Science. (2020) 367:405–11. doi: 10.1126/science.aax0249, PMID: 31974247 PMC7694873

[25] Jin S Plikus MV Nie Q. CellChat for systematic analysis of cell-cell communication from single-cell transcriptomics. Nat Protoc. (2025) 20:180–219. doi: 10.1038/s41596-024-01045-4, PMID: 39289562

[26] Xu S Hu E Cai Y Xie Z Luo X Zhan L. Using clusterProfiler to characterize multiomics data. Nat Protoc. (2024) 19:3292–320. doi: 10.1038/s41596-024-01020-z, PMID: 39019974

[27] Elosua-Bayes M Nieto P Mereu E Gut I Heyn H. SPOTlight: seeded NMF regression to deconvolute spatial transcriptomics spots with single-cell transcriptomes. Nucleic Acids Res. (2021) 49:e50. doi: 10.1093/nar/gkab043, PMID: 33544846 PMC8136778

[28] Liu Q Hsu CY Shyr Y. Scalable and model-free detection of spatial patterns and colocalization. Genome Res. (2022) 32:1736–45. doi: 10.1101/gr.276851.122, PMID: 36223499 PMC9528978

[29] Filippou PS Karagiannis GS Constantinidou A. Midkine (MDK) growth factor: a key player in cancer progression and a promising therapeutic target. Oncogene. (2020) 39:2040–54. doi: 10.1038/s41388-019-1124-8, PMID: 31801970

[30] Münter D de Faria FW Richter M Aranda-Pardos I Hotfilder M Walter C. Multiomic analysis uncovers a continuous spectrum of differentiation and Wnt-MDK-driven immune evasion in hepatoblastoma. J Hepatol. (2025) 83:367–82. doi: 10.1016/j.jhep.2025.01.031, PMID: 39900120

[31] Zhang Y Zuo C Liu L Hu Y Yang B Qiu S. Single-cell RNA-sequencing atlas reveals an MDK-dependent immunosuppressive environment in ErbB pathway-mutated gallbladder cancer. J Hepatol. (2021) 75:1128–41. doi: 10.1016/j.jhep.2021.06.023, PMID: 34171432

[32] Zhao Y Chen C Chen K Sun Y He N Zhang X. Multi-omics analysis of macrophage-associated receptor and ligand reveals a strong prognostic signature and subtypes in hepatocellular carcinoma. Sci Rep. (2024) 14:12163. doi: 10.1038/s41598-024-62668-x, PMID: 38806553 PMC11133315

[33] Ye B Hongting G Zhuang W Chen C Yi S Tang X. Deciphering lung adenocarcinoma prognosis and immunotherapy response through an AI-driven stemness-related gene signature. J Cell Mol Med. (2024) 28:e18564. doi: 10.1111/jcmm.18564, PMID: 39046884 PMC11268368

[34] Ye B Ji H Zhu M Wang A Tang J Liang Y. Single-cell sequencing reveals novel proliferative cell type: a key player in renal cell carcinoma prognosis and therapeutic response. Clin Exp Med. (2024) 24:167. doi: 10.1007/s10238-024-01424-x, PMID: 39052149 PMC11272756

[35] Yuan F Wang Y Yuan L Tang T Ye L Li Y. EGFRvIII-positive glioblastoma contributes to immune escape and Malignant progression via the c-Fos-MDK-LRP1 axis. Cell Death Dis. (2025) 16:453. doi: 10.1038/s41419-025-07771-1, PMID: 40527884 PMC12174314

[36] Ye B Li Z Wang Q. A novel artificial intelligence network to assess the prognosis of gastrointestinal cancer to immunotherapy based on genetic mutation features. Front Immunol. (2024) 15:1428529. doi: 10.3389/fimmu.2024.1428529, PMID: 38994371 PMC11236566

[37] Stip MC Teeuwen L Dierselhuis MP Leusen JHW Krijgsman D. Targeting the myeloid microenvironment in neuroblastoma. J Exp Clin Cancer Res. (2023) 42:337. doi: 10.1186/s13046-023-02913-9, PMID: 38087370 PMC10716967

[38] Cerezo-Wallis D Contreras-Alcalde M Troulé K Catena X Mucientes C Calvo TG. Midkine rewires the melanoma microenvironment toward a tolerogenic and immune-resistant state. Nat Med. (2020) 26:1865–77. doi: 10.1038/s41591-020-1073-3, PMID: 33077955

[39] Li S Yu J Huber A Kryczek I Wang Z Jiang L. Metabolism drives macrophage heterogeneity in the tumor microenvironment. Cell Rep. (2022) 39:110609. doi: 10.1016/j.celrep.2022.110609, PMID: 35385733 PMC9052943

[40] Wu Z Liu D Ou Y Xu Z Heng G Liu W. Mechanism and endoscopic-treatment-induced evolution of biliary non-anastomotic stricture after liver transplantation revealed by single-cell RNA sequencing. Clin Transl Med. (2024) 14:e1622. doi: 10.1002/ctm2.1622, PMID: 38481381 PMC10938070

[41] Yang Q Zhang H Wei T Lin A Sun Y Luo P. Single-cell RNA sequencing reveals the heterogeneity of tumor-associated macrophage in non-small cell lung cancer and differences between sexes. Front Immunol. (2021) 12:756722. doi: 10.3389/fimmu.2021.756722, PMID: 34804043 PMC8602907

[42] Liu X Qin J Nie J Gao R Hu S Sun H. ANGPTL2+cancer-associated fibroblasts and SPP1+macrophages are metastasis accelerators of colorectal cancer. Front Immunol. (2023) 14:1185208. doi: 10.3389/fimmu.2023.1185208, PMID: 37691929 PMC10483401

[43] Su W Ye Z Liu J Deng K Liu J Zhu H. Single-cell and spatial transcriptome analyses reveal tumor heterogeneity and immune remodeling involved in pituitary neuroendocrine tumor progression. Nat Commun. (2025) 16:5007. doi: 10.1038/s41467-025-60028-5, PMID: 40442104 PMC12122724

[44] Sathe A Mason K Grimes SM Zhou Z Lau BT Bai X. Colorectal cancer metastases in the liver establish immunosuppressive spatial networking between tumor-associated SPP1+ Macrophages and fibroblasts. Clin Cancer Res. (2023) 29:244–60. doi: 10.1158/1078-0432.CCR-22-2041, PMID: 36239989 PMC9811165

[45] Liu Y Zhang Q Xing B Luo N Gao R Yu K. Immune phenotypic linkage between colorectal cancer and liver metastasis. Cancer Cell. (2022) 40:424–37.e5. doi: 10.1016/j.ccell.2022.02.013, PMID: 35303421

[46] Matusiak M Hickey JW van IDGP Lu G Kidziński L Zhu S. Spatially segregated macrophage populations predict distinct outcomes in colon cancer. Cancer Discov. (2024) 14:1418–39. doi: 10.1158/2159-8290.CD-23-1300, PMID: 38552005 PMC11294822

[47] Hong SM Lee AY Kim BJ Lee JE Seon SY Ha YJ. NAMPT-driven M2 polarization of tumor-associated macrophages leads to an immunosuppressive microenvironment in colorectal cancer. Adv Sci (Weinh). (2024) 11:e2303177. doi: 10.1002/advs.202303177, PMID: 38308188 PMC11005718

[48] Xu C Sun L Jiang C Zhou H Gu L Liu Y. SPP1, analyzed by bioinformatics methods, promotes the metastasis in colorectal cancer by activating EMT pathway. BioMed Pharmacother. (2017) 91:1167–77. doi: 10.1016/j.biopha.2017.05.056, PMID: 28531945

[49] Wang Y Wang Q Tao S Li H Zhang X Xia Y. Identification of SPP1(+) macrophages in promoting cancer stemness via vitronectin and CCL15 signals crosstalk in liver cancer. Cancer Lett. (2024) 604:217199. doi: 10.1016/j.canlet.2024.217199, PMID: 39216547

[50] Zhang Q Liu Y Wang X Zhang C Hou M Liu Y. Integration of single-cell RNA sequencing and bulk RNA transcriptome sequencing reveals a heterogeneous immune landscape and pivotal cell subpopulations associated with colorectal cancer prognosis. Front Immunol. (2023) 14:1184167. doi: 10.3389/fimmu.2023.1184167, PMID: 37675100 PMC10477986

[51] Craig SG Humphries MP Alderdice M Bingham V Richman SD Loughrey MB. Immune status is prognostic for poor survival in colorectal cancer patients and is associated with tumour hypoxia. Br J Cancer. (2020) 123:1280–8. doi: 10.1038/s41416-020-0985-5, PMID: 32684627 PMC7555485

[52] Ye B Fan J Xue L Zhuang Y Luo P Jiang A. iMLGAM: Integrated Machine Learning and Genetic Algorithm-driven Multiomics analysis for pan-cancer immunotherapy response prediction. Imeta. (2025) 4:e70011. doi: 10.1002/imt2.70011, PMID: 40236779 PMC11995183

