# Supplementary material for: Single-cell and spatial transcriptomics integration reveals FAM49B promotes tumor-associated macrophages polarization in colorectal cancer via the MK pathway
Source: Front Immunol. 2025 Oct 31;16:1682637. doi: 10.3389/fimmu.2025.1682637 (PMC12615435; doi:10.3389/fimmu.2025.1682637)
Supplement: Supplementary file 1 [file Image1.pdf]

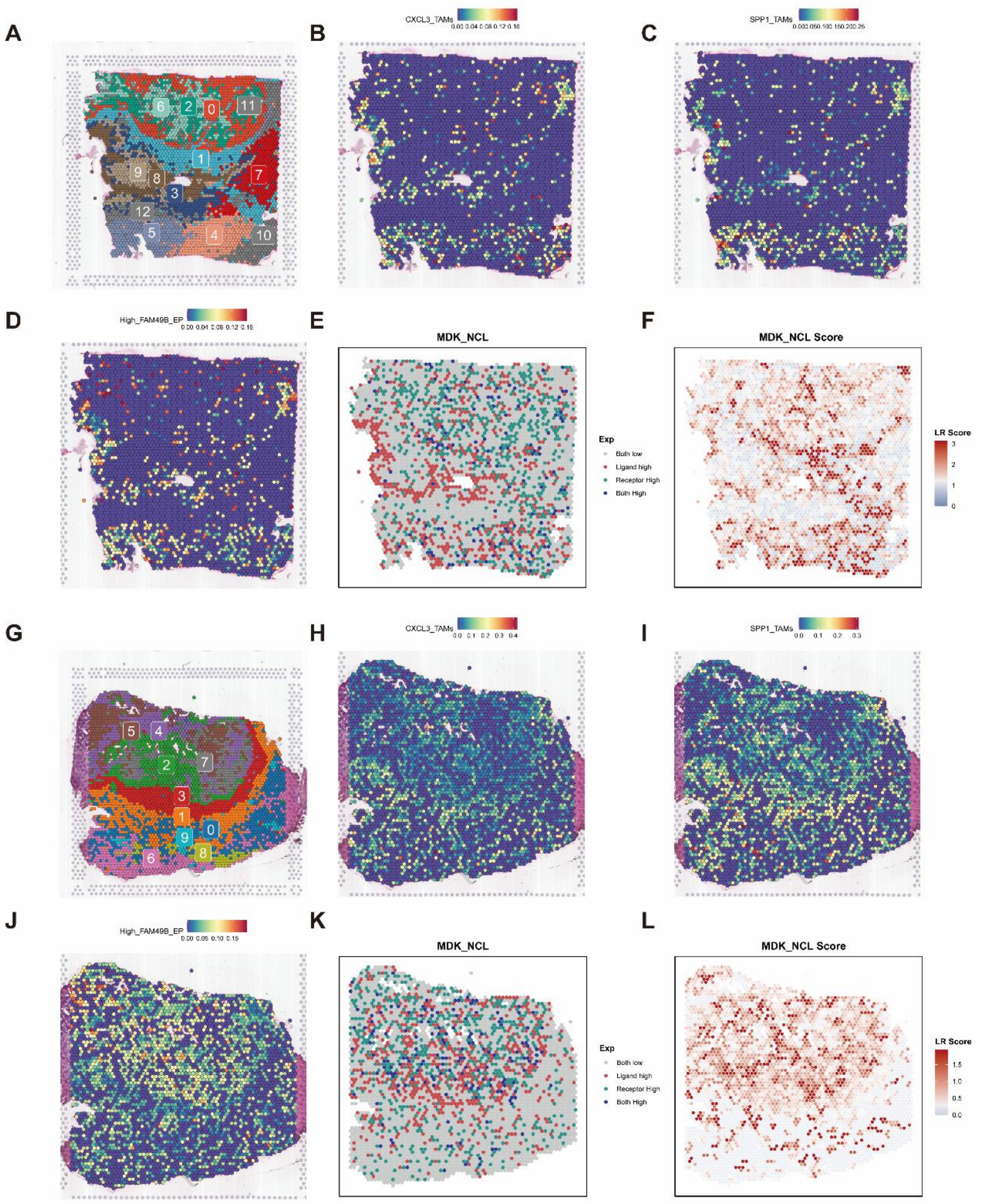

Supplementary Figure 1. Spatial transcriptomic slices of primary colorectal cancer lesions. (A) Cluster plot of 0-12 subgroups clustered by Seurat. (B-D) Spatial plot showing the expression of CXCL3_TAMs, SPP1_TAMs, and High_FAM49B_EP in PT predicted by SPOTlight. (E, F) Spatial mapping of the MDK ligand, NCL receptor, and their binding score in the MDK-NCL ligand-receptor interaction analysis in PT. (G) Cluster plot of 0-9 subgroups clustered by Seurat. (H-J) Spatial plot showing the expression of CXCL3_TAMs, SPP1_TAMs, and High_FAM49B_EP in

LM predicted by SPOTlight. (K, L) Spatial mapping of the MDK ligand, NCL receptor, and their binding score in the MDK-NCL ligand-receptor interaction analysis in LM.
